# A Transgenic mouse tolerant to syngeneic cancer cells expressing truncated human epidermal growth factor receptor

**DOI:** 10.1371/journal.pone.0355841

**Published:** 2026-08-07

**Authors:** Theresa Barberi, Rahila Khuroo, Alan D. Friedman

**Affiliations:** Division of Pediatric Oncology, Johns Hopkins University School of Medicine, Baltimore, Maryland, United States of America; University of Tennessee Health Science Center, UNITED STATES OF AMERICA

## Abstract

Epidermal Growth Factor Receptor (EGFR) is often present on the cell surface of a wide variety human malignancies, including non-small-cell lung cancer (NSCLC), glioblastoma (GBM), pancreatic ductal carcinoma (PDC), and castration-resistant prostate cancer (CRPC). Efforts to optimize immunotherapies targeting EGFR are limited by murine intolerance of human EGFR. To overcome this obstacle, we developed C57BL/6 mice in which a truncated variant of human EGFR (hEGFRt), lacking the ligand binding domain and cytoplasmic domain, is expressed from the CAG regulatory elements comprised of the CMV enhancer, β-actin promoter, and β-globin poly-adenylation signals. Cetuximab, a high-affinity, clinically available anti-human EGFR antibody, retains affinity for hEGFRt. The hEGFRt cDNA in the CAG-hEGFRt transgene is flanked by *lox*P sites to enable its excision thereby reducing interaction of hEGFRt-directed immunotherapies with normal tissues. The CAG-hEGFRt(f/f) transgene is abundantly expressed in hematopoietic lymphoid and myeloid cells, with low-level expression evident also in non-hematopoietic liver, lung, kidney, and brain. Mx1-Cre-mediated transgene excision in adult mice reduces hEGFRt ~ 5-fold in blood mononuclear cells. CAG-hEGFRt(f/f) adult mice are tolerant of syngeneic NSCLC, GBM, prostate, and PDC lines expressing hEGFRt. These mice retain hEGFRt tolerance after transgene deletion. CAG-hEGFRt(f/f) mice provide a new and important tool for the development of immunotherapies targeting hEGFR.

## Introduction

Human EGFR (hEGFR) is a highly relevant immunotherapy target, as it is often expressed on non-small-cell lung cancer (NSCLC), glioblastoma (GBM), castration-resistant prostate cancer (CRPC), pancreatic ductal carcinoma (PDC), triple-negative breast cancer (TNBC), colorectal carcinoma (CRC), and other human malignancies [1–7]. Approximately 60% of GBMs over-express EGFR; additionally, the EGFR gene frequently contains activating mutations, such as the activated EGFRvIII splice variant that lacks the ligand-binding domain, which occurs in ~10% of GBM cases [7,8]. EGFR is also expressed on 41% of newly diagnosed prostate cancers, 76% of CRPCs, and 100% of metastatic prostate cancers [4]. Nearly 75% of NSCLC cases express EGFR, and a subset of these have targetable, activating mutations [1,9].

We are investigating the efficacy of adoptively transferred immature myeloid cells rendered pro-inflammatory due to absence of the repressive NF-κB p50 subunit (p50-IMC) as a novel cancer immunotherapy. We observe responses in syngeneic murine prostate cancer, PDC, and neuroblastoma models, dependent on activation of anti-tumor T cell immunity [10, 11]. p50-IMC complexed with anti-human EGFR antibody (Cetuximab) or anti-human PSMA antibody manifest increased localization to prostate cancer tumors expressing either human EGFR or PSMA, in immune-deficient NSG mice. Additionally, expression of a PSMA chimeric antigen receptor (CAR) on p50-IMC also increases tumor localization in NSG mice [12]. However, our ability to investigate hEGFR-directed p50-IMC efficacy in immune-competent mice was prevented by their intolerance to hEGFR. Myc-CaP prostate cancer cells expressing surface hEGFR formed large tumors that retained hEGFR expression after subcutaneous (sq) inoculation into NSG mice; conversely, 6 of 7 syngeneic FVBN mice never developed tumors even after 90 days post-inoculation, and the one small tumor that did grow lacked hEGFR expression [12]. Cetuximab does not interact with murine EGFR, precluding anti-tumor efficacy studies with cancer cells expressing murine EGFR. Monoclonal antibody 7A7 was initially reported to interact with murine EGFR, but later studies disputed this claim [13].

To assist evaluation and optimization of immunotherapies targeting hEGFR, we developed CAG-hEGFRt(f/f) mice. The hEGFRt transgene lacks the extra-cellular ligand-binding domain and the intra-cellular signaling domain and is therefore expected to be functionally inert. A similar hEGFRt construct was utilized as a selection epitope in the context of a lentiviral vector, with hEGFRt retaining affinity for Cetuximab and having no effect on T cell expression of surface CD3, CD4, CD8, CD28, TCRα/β, or Granzyme A, T cell expansion, or T cell production of IFNγ or TNFα [14]. *Lox*P sites surrounding the hEGFRt transgene in the mice we developed allow its Cre-mediated deletion in adult mice, of potential utility to reduce off-target effects of hEGFR-directed cancer immunotherapies. Tumors that develop from syngeneic NSCLC, GBM, PDC, and prostate cancer lines in CAG-hEGFRt(f/f) mice retain expression of exogenous, cell surface hEGFRt.

## Materials and methods

### Ethics statement

This study was carried out in strict accordance with the recommendations in the Guide for the Care and Use of Laboratory Animals of the National Institutes of Health. The protocols were approved by the Johns Hopkins University Animal Care and Use Committee (MO21M390, MO24M280). All efforts were made to minimize suffering. As procedures caused only momentary, slight pain, analgesics and anesthesia were not required. Euthanasia prior to tumor harvest was by carbon dioxide asphyxiation, followed by cervical dislocation.

### Transgenic mouse generation

A DNA segment having two *lox*P sites and surrounding restriction sites was synthesized in the context of a pUC19 vector (Blue Heron Biotech), as follows: *Eco*R1 - *lox*P - *Kpn*I – BglII – *Not*I – *Nhe*I – *Mlu*I – *Xho*I - *lox*P - *Bam*HI. A standard *lox*P site has an internal 8 bp spacer, 5’-ATGTATGC. To avoid presence of a 5’-ATG sequence that might be used to aberrantly initiate translation, we utilized *lox*P sites in which this 8 bp segment (underlined) is inverted:

*lox*P sites: 5’- ATAACTTCGTATAGCATACATTATACGAAGTTAT-3’.

A murine codon-optimized cDNA encoding amino acids 1−22 of the human GM-CSF Receptor (GMCSFR) α chain followed by hEGFR residues 310−646 and a stop codon, flanked by *Not*I and *Xho*I sites, was synthesized (Blue Heron Biotech). The GMCSFR segment provides a leader polypeptide signal sequence to direct the translated protein to the plasma membrane. A DNA segment containing Kozak’s rules (KR) for optimal ribosomal initiation, 5’-GCCGCCACC-3’, was positioned just upstream of the initiating ATG. The *Not*I/*Xho*I segment containing the KR-leader polypeptide-hEGFRt sequences was transferred between the *lox*P sites in the pUC19-*lox*P plasmid using directional cloning. For simplicity, we refer to the GMCSFR leader peptide-hEGFRt combination as hEGFRt. An *Eco*RI/*Bam*HI segment contained the *lox*P-site flanked hEGFRt cDNA, hEGFRt(f/f), was inserted into *Eco*R1/*Bgl*II digested pCAGGS-mCherry (Addgene, #41583), replacing the mCherry cDNA. The full sequence of this insert is provided (S1 Fig.). The CAG-hEGFRt(f/f) segment, in which the CMV enhancer/chicken β-actin promoter and rabbit β-globin poly-adenylation signals flank the hEGFRt(f/f) cDNA, was released from vector sequences by SalI/*Bam*HI digestion, isolated using a QIAquick Gel Extraction Kit (Qiagen) after agarose gel electrophoresis, and provided to the Johns Hopkins Transgenic Core facility for micro-injection into C57BL/6 (B6) blastocysts. Two founders were identified from 27 P0 offspring screened by tail clip DNA PCR (38 cycles, 95^o^C 30 sec, 60^o^C 30 sec, 72^o^C 30 sec) using primers:

CAG-F1: 5’-CTGTCTCATCATTTTGGCAAAG-3’ and EGFR-34R: 5’-CAGCAGGAACCCTGTGATC-3’, yielding a 401 bp product. One of these founders provided germline transmission to subsequent generations and has been deposited in Jackson Laboratories (Stock No. 040502).

### Cell culture and transduction

The hEGFR and hEGFRt(f/f) cDNAs were inserted into the polylinker of the MIPuro retroviral vector. B6-derived Lewis Lung Carcinoma (LLC) cells (ATCC, CRL-1642), GL261-Luciferase (Luc) GBM cells [15], RM-1 prostate cancer cells (ATCC, CRL-3310), and UN-KC-6141 PDC cells [10], as well as 293T human embryonic kidney cells (ATCC, CRL-3216) were maintained in Dulbecco’s modified Eagle medium (DMEM) with 10% heat-inactivated fetal bovine serum (HI-FBS) and antibiotic/antimycotic (AA, Sigma). Retroviral vectors were packaged by transfection with pkat2ecopac into 293T cells using Lipofectamine 2000 [16]. LLC, GL261-Luc, PDC, and RM-1 cells were transduced using 293T supernatant and Polybrene (4 µg/mL), followed by puromycin selection.

### Tumor growth

B6 mice were obtained from Charles River Laboratories (#556). NSG mice (#5557) and B6-derived Mx1-Cre mice (#3556) were obtained from Jackson Laboratories. 3E5 LLC/hEGFR, 3E5 LLC/hEGFRt, 3E6 PDC, or 2E6 RM-1 cells were suspended in 100 µL Hanks’ balanced salt solution (HBSS) and inoculated sq into the mouse flank. 8- to 16-week-old male and female mice were utilized. Tumor sizes were monitored using calipers. Tumor volumes were estimated as length x width-squared divided by two. 5E4 GL261-Luc/hEGFRt cells were inoculated intra-cranially, as described [15]. Polyinosinic:polycytidylic (pIpC) high molecular weight double-stranded RNA (InvivoGen, catalog #tlrl-pic) was provided as 300 µg intra-peritoneal injections on days 0, 2, and 4 to induce Mx1-Cre expression.

### Flow cytometry

Tumors and organs from euthanized mice were dissociated into single cells using collagenase, hyaluronidase, Dispase, and DNaseI (StemCell Technologies) and then passed through a 40 µM cell strainer as described [11]. Marrow flushed from the long bones and peripheral blood obtained by facial vein lancing was subjected to red blood cell lysis using ammonium chloride. Thymus was dissociated into single cells by mechanical crushing and passage through a 40 µm cell strainer. For flow cytometry, after blocking with anti-FcγR2/FcγR3 antibody (Ab) for 15 minutes, fluorochrome-conjugated Abs were added for 45 minutes. Cells were analyzed using an LSR Fortessa Flow Cytometer (BD Biosciences), first gating on live cells that exclude propidium iodide (BioLegend). Next, CD45-negative cells were gated for analysis of liver, lung, kidney, and brain (S2 Fig.). Antibodies used were anti-hEGFR-APC, Cetuximab followed by ant-human IgG-PE anti-CD45-BV421, anti-CD19-BV421, anti-CD3-FITC, anti-CD4-PE, anti-CD8-BV650, anti-CD11b-BV421, and anti-Ter119-PE. All antibodies were from BioLegend.

### Statistics

Means and standard deviations (SD) are shown. The Student’s t test was used for statistical comparisons.

## Results

### Generation of CAG-hEGFRt(f/f) transgenic mice

**Fig 1:**
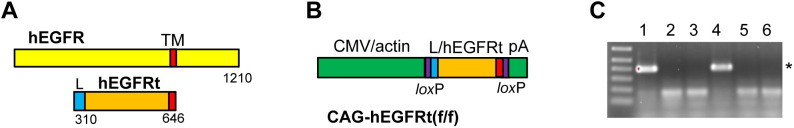
Generation of CAG-hEGFRt(f/f) transgenic mice. **A**) Diagram of hEGFR (top) and hEGFRt (bottom). TM – transmembrane domain; L – leader peptide. **B**) Diagram of the CAG-hEGFRt(f/f) transgene. CAG – CMV enhancer, actin promoter, β-globin polyadenylation (pA) signal; f/f – flox/flox. **C**) Tail clip DNAs from P0 pups 1-6 were subjected to PCR to detect the transgene DNA (*), followed by agarose gel electrophoresis and ethidium bromide staining.

A diagram of hEGFR and truncated hEGFRt is shown (Fig 1A). We utilized a floxed version of the hEGFRt transgene to allow for more precise expression that could better reflect the human expression pattern of hEGFR. CAG-hEGFRt(f/f) transgene DNA (Fig 1B) was provided, vector-free, to the Johns Hopkins Transgenic core, which performed B6 blastocyst micro-injections. Two of 27 offspring screened had transgene DNA detected in tail clip DNA, and one of these transmitted the transgene to their offspring (Fig 1C), establishing a transgenic line.

**Fig 2:**
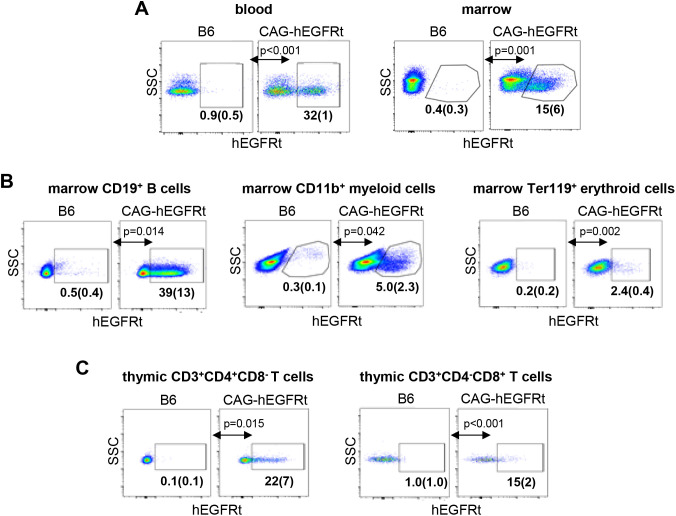
Expression of hEGFRt on hematopoietic cells from CAG-hEGFRt(f/f) mice. **A**) hEGFRt surface expression was assessed by flow cytometry on nucleated blood cells and total marrow mononuclear cells from parental B6 or CAG-hEGFRt(f/f) transgenic mice. **B, C**) hEGFRt surface expression was assessed on marrow CD19^+^ B cells, CD11b^+^ myeloid cells, Ter119^+^ erythroid cells and on thymic CD3^+^CD4^+^CD8^-^ and CD3^+^CD4^-^CD8^+^ mature T cells. SSC – side scatter. (B6 n = 3, hEGFRt(f/f) n = 3; mean, SD, and p-values are shown, with SD values in parentheses).

**Fig 3:**
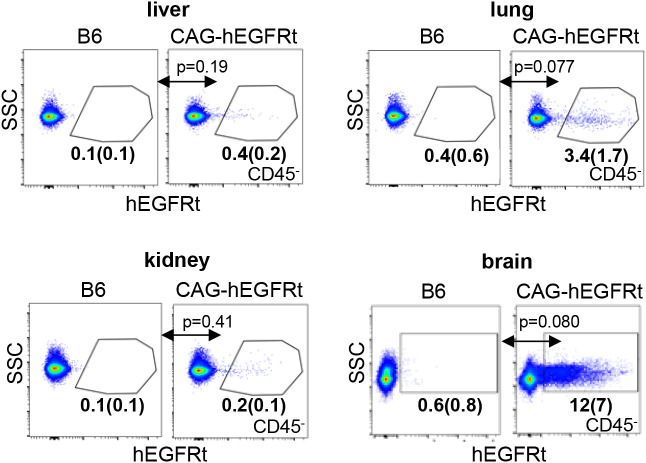
Expression of hEGFRt on non-hematopoietic cells in CAG-hEGFRt(f/f) mice. hEGFRt surface expression was assessed by flow cytometry on CD45- single-cell suspensions of liver, lung, kidney, and brain, from parental B6 or CAG-hEGFRt(f/f) transgenic mice. SSC – side scatter (B6 n = 3, hEGFRt(f/f) n = 3; mean, SD, and p-values are values shown).

To characterize the expression of hEGFRt in our trangenic line, we isolated hematopoietic tissues and several organs, stained these with anti-hEGFR and other antibodies, and then conducted flow cytometry. The pan-hematopoietic surface marker CD45 was used to distinguish between hematopoietic and non-hematopoietic cells. We observed that, on average, 32% of nucleated blood cells and 15% of total marrow mononuclear cells express surface hEGFRt (Fig 2A). Within the bone marrow, we found hEGFRt present on 39% of CD19^+^ B cells, 5.0% of CD11b^+^ myeloid cells, and 2.4% of Ter119^+^ erythroid progenitors/precursors (Fig 2B). Within the thymus, 20% of CD3^+^CD4^+^CD8^-^ and 11% of CD3^+^CD4^-^CD8^+^ mature T cells expressed hEGFRt (Fig 2C). In addition, 3% of CD3^-^CD4^+^CD8^+^and 15% of CD3^+^CD4^+^CD8^+^ T cell precursors expressed hEGFRt (not shown). We also evaluated non-hematopoietic expression of hEGFRt in several organs, finding 0.4% of liver cells, 3.4% of lung cells, 0.2% of kidney cells, and 12% of brain cells to be hEGFRt^+^, with a trend towards increased expression above background hEGFRt flow signal in lung and brain cells (Fig 3). Together, these data establish that adult hEGFRt(f/f) mice express hEGFRt, with expression most abundant on hematopoietic lymphoid and myeloid cells.

### CAG-hEGFRt(f/f) mice tolerate surface expression of hEGFRt on malignant tumors

We initially expressed full-length hEGFR in the LLC cell line, which was confirmed by flow cytometry (S3A Fig.). LLC/hEGFR cells readily formed sq tumors in immune-deficient NSG mice, and the tumors retained hEGFR expression; when inoculated into syngeneic wild-type (WT) B6 mice, however, only a few small tumors grew out, and they did not retain hEGFR expression (S3B Fig.). No LLC/hEGFR tumors formed in CAG-hEGFRt(f/f) mice (not shown).

**Fig 4:**
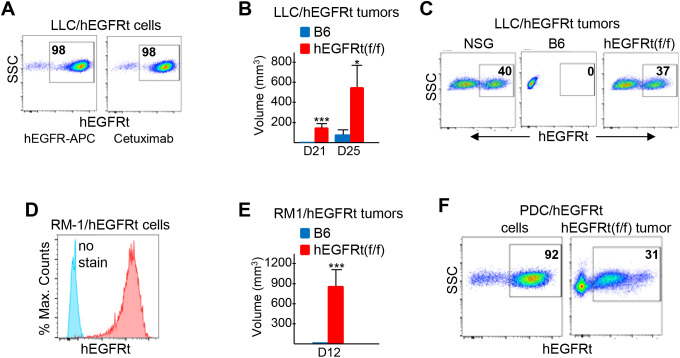
CAG-hEGFRt(f/f) mice tolerate hEGFRt expression. **A**) LLC NSCLC cells transduced with MIPuro-hEGFRt and subjected to puromycin selection were evaluated for cell surface hEGFRt expression by flow cytometry, using either anti-hEGFR-APC antibody (left) or Cetuximab and anti-human IgG-PE (right). **B**) LLC/hEGFRt cells were injected sq into parental B6 (n = 10) or CAG-hEGFRt(f/f) (n = 6) mice and tumor sizes were evaluated on day 21 (D21) and day 25 (D25). *p < 0.05, ***p < 0.001. **C**) LLC/hEGFRt tumors obtained in NSG, B6, or CAG-hEGFRt(f/f) mice were evaluated for hEGFRt expression. **D**) RM-1 prostate cancer cells transduced with MIPuro-hEGFRt and subjected to puromycin selection were evaluated for hEGFRt expression. **E**) RM-1/hEGFRt cells were injected sq into parental B6 (n = 3) or CAG-hEGFRt(f/f) (n = 4) male mice and tumor sizes were evaluated on day 12. ***p < 0.001. **F**) hEGFRt expression on PDC/hEGFRt cells and a tumor obtained after these cells were injected sq into a CAG-hEGFRt(f/f) mouse.

We next transduced the LLC cell line with truncated hEGFRt. We confirmed expression by flow cytometry using both a commercially available anti-hEGFR-APC antibody or Cetuximab followed by an anti-human IgG secondary Ab (Fig 4A). LLC/hEGFRt cells were inoculated sq into syngeneic WT B6 mice and CAG-hEGFRt(f/f) transgenic mice which were then followed for tumor growth. After 21 days, WT B6 mice still did not have measurable tumors, whereas hEGFRt(f/f) mice had visible tumors averaging ~200 mm^3^. By day 25, tumors had appeared on most WT mice, but they were ~6-fold smaller in volume, on average, than those of hEGFRt(f/f) mice (Fig 4B). We isolated tumors from both WT and hEGFRt(f/f) mice to evaluate whether hEGFRt expression was retained in the tumors that formed. Tumors from immune-deficient NSG mice were included as a positive control for retained hEGFRt expression. We found that hEGFRt expression persisted in LLC/hEGFRt tumors from control NSG mice and CAG-hEGFRt(f/f) mice, but not in WT B6 mice (Fig 4C), indicating that the outgrowth of larger tumors in WT mice had resulted from the selection of a small subset of the LLC/hEGFRt cell population that lacked or lost hEGFRt expression.

We evaluated several additional tumor models for the expression and retention of hEGFRt. RM-1 prostate cancer cells transduced with hEGFRt express abundant hEGFRt surface protein (Fig 4D) and readily form sq tumors in CAG-hEGFRt(f/f) mice but display only minimal tumor growth in WT B6 mice (Fig 4E). Pancreatic ductal carcinoma (PDC) cells expressing abundant hEGFRt also form sq tumors that retain hEGFRt expression in CAG-hEGFRt(f/f) mice (Fig 4F). The GL261 glioblastoma line was also successfully transduced with hEGFRt, and hEGFRt expression was retained in a brain tumor isolated from a CAG-hEGFRt(f/f) but not a parental B6 mouse (S4 Fig.).

Overall, these experiments demonstrate tolerance of hEGFRt expression in four different cancer lines by CAG-hEGFRt(f/f) mice.

### Mx1-Cre reduces hEGFRt expression with retention of tolerance in adult transgenic mice

**Fig 5:**
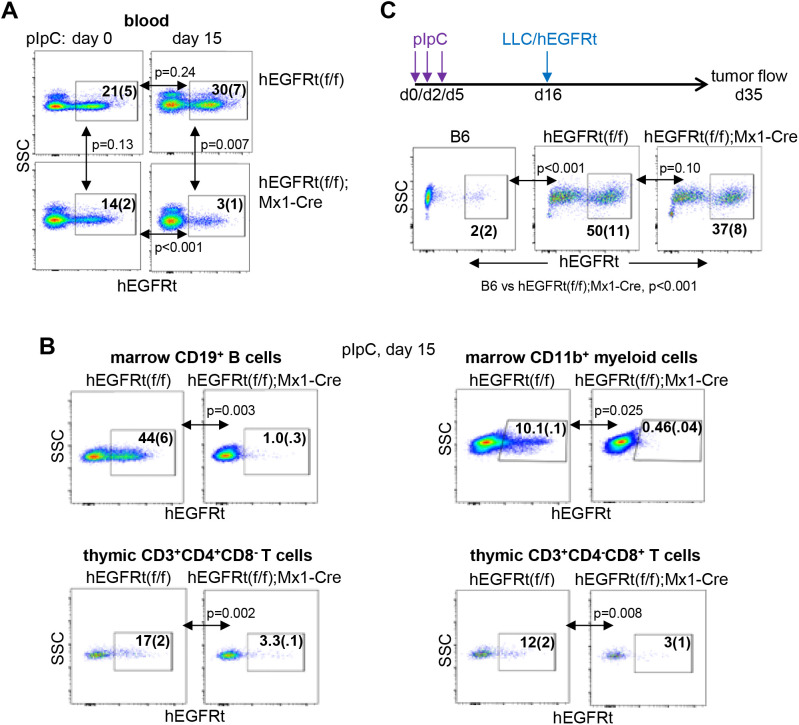
Deletion of the hEGFRt(f/f) transgene in adult mice does not disrupt hEGFRt tolerance. **A)** CAG-hEGFRt(f/f) and CAG-hEGFRt(f/f);Mx1-Cre mice received pIpC on days 0, 2, and 4. Peripheral blood mononuclear cells were analyzed for hEGFRt expression on day 0 and day 15 via flow cytometry (n = 3; mean, SD, and p-values are shown). **B**) Bone marrow CD19^+^ B lymphoid and CD11b^+^ myeloid cells and thymic CD4^+^ and CD8^+^ T cells were analyzed for hEGFRt expression on day 15 (n = 3; mean, SD, and p-values are shown). **C**) LLC/hEGFRt cells were injected sq on day 16 into B6 (n = 5), CAG-hEGFRt(f/f) (n = 3), and CAG-hEGFRt(f/f);Mx1-Cre (n = 5) mice that had received pIpC on days 0, 2, and 5, followed by analysis for tumor cell surface hEGFRt on day 35, as diagrammed (top). Representative flow cytometry data are shown (bottom, with mean, SD, and p-values).

We next evaluated tolerance to tumor hEGFRt after Mx1-Cre mediated deletion of hEGFRt in adult mice. CAG-hEGFRt(f/f) and CAG-hEGFRt(f/f);Mx1-Cre mice received pIpC on days 0, 2, and 4. pIpC double-stranded RNA induces interferons, which in turn activate the Mx1 promoter and thereby Cre expression. To confirm successful Cre-mediated deletion, hEGFRt expression on nucleated blood cells was analyzed on day 0 and on day 15 after pIpC (Fig 5A). On day 0, we observed a trend towards a reduced proportion of blood cells expressing hEGFRt (average 21% in CAG-hEGFRt(f/f) versus 14% in CAG-hEGFRt(f/f);Mx1-Cre, p = 0.13), presumably due to leaky Mx1-Cre expression. Day 15 analysis showed that pIpC successfully reduced hEGFRt expression in CAG-hEGFRt(f/f);Mx1-Cre mice (average 14% in CAG-hEGFRt(f/f);Mx1-Cre before pIpC versus 3% after pIpC, p < 0.001) but did not reduce the proportion of cells expressing hEGFRt in CAG-hEGFRt(f/f) mice (average 21% in CAG-hEGFRt(f/f) before pIpC versus 30% after pIpC). Overall, Mx1-Cre induction reduced blood hEGFRt expression ~7-fold. pIpC administration to CAG-hEGFRt(f/f);Mx1-Cre mice also significantly reduced hEGFRt expression in marrow CD19^+^ B cells, CD11b+ myeloid cells, and thymic CD4^+^ and CD8^+^ T cells by an average of 44-, 22-, 5-, and 4-fold, respectively (Fig 5B).

We confirmed that hEGFRt tolerance was retained by inoculating pIpC-injected B6, CAG-hEGFRt(f/f), and CAG-hEGFRt(f/f);Mx1-Cre mice with LLC/hEGFRt cells, followed by flow cytometry for hEGFRt on tumor cells, as diagrammed (Fig 5C**, top**). hEGFRt was expressed, on average, on 2% of tumor cells in B6 mice, 50% of tumor cells in CAG-hEGFRt(f/f), and 37% of tumor cells in CAG-hEGFRt(f/f);Mx1-Cre mice (Fig 5C**, bottom**). The difference in hEGFRt expression on LLC tumor cells between hEGFRt(f/f) and hEGFRt(f/f);Mx1-Cre mice was not statistically significant. These data indicate that Cre-expression reduces hEGFRt expression in adult CAG-hEGFRt(f/f) mice but that these mice retain tolerance to cell surface hEGFRt.

## Discussion

A wide variety of aggressive cancers express hEGFR or activated variants, making hEGFR a promising target for cancer immunotherapy. Such therapies might include T cells, NK cells, or myeloid cells expressing CARs or having surface bound antibodies. Mice lack tolerance for full-length hEGFR [12], and data herein demonstrate that mice also lack tolerance for truncated hEGFRt, in which the 309 amino acid hEGFR extra-cellular ligand binding domain and the 564-residue cytoplasmic domain have been deleted. As a result, evaluation of immunotherapies targeting EGFR in a fully immune-competent animal model has not been possible.

To address this problem, we developed CAG-hEGFRt(f/f) transgenic mice. The same CAG regulatory elements were used to create a mouse line tolerant to human mesothelin [17]. CAG-hEGFRt(f/f) mice express hEGFRt most abundantly on hematopoietic lymphoid and myeloid cells and to a lesser extent on non-hematopoietic cells in multiple solid organs. Although hEGFRt mice do not tolerate full-length hEGFR, they do tolerate hEGFRt expressed on the surface of the several syngeneic B6-derived cancer cell lines we evaluated, representing NSCLC, GBM, prostate cancer, and PDC.

The hEGFRt cDNA in CAG-hEGFRt(f/f) mice is flanked by *lox*P sites. Cre expression, induced from the Mx1 promoter by pIpC in adult mice, reduces hEGFRt expression on nucleated blood cells by ~7-fold while maintaining tolerance to tumor hEGFRt. Reduced expression of hEGFRt on hematopoietic and other cells might better mimic the extent of exogenous hEGFR expression in humans, providing a more accurate model for assessing hEGFR-directed immunotherapy efficacy and toxicity.

CAG-hEGFRt(f/f) mice are available from Jackson Laboratory (Stock No. 040502).

## Supporting information

S1 Fig Sequence of floxed hEGFRt(f/f) transgene. Synthesized DNA sequence that includes the hEGFRt transgene with an N-terminal GM-CSFR leader peptide, a Kozak sequence to favor ribosomal initiation, and flanking restriction enzyme and *lox*P sites.
(TIF)

S2 Fig Flow cytometry gating of hematopoietic and non-hematopoietic cells in tissues from CAG-hEGFRt(f/f) mice. A) Marrow, kidney, liver, lung, or thymus single-cell suspensions were gated based on FSC/SSC characteristics to separate cells from debris, followed by propidium iodide (PI) staining to identify viable cells, and then CD45 staining to identify CD45^+^ hematopoietic and CD45^-^ non-hematopoietic cells. **B**) Brain single-cell suspensions were gated similarly. Representative flow cytometry data is shown. Cells were subsequently stained for hEGFRt expression (see Figs. 2, 3). FSC – forward scatter; SSC – side scatter.
(TIF)

S3 Fig Parental C57BL/6 mice do not tolerate hEGFR. A) LLC cells transduced with MIPuro-hEGFR and subjected to puromycin selection were evaluated for cell surface hEGFR expression by flow cytometry using anti-hEGFR-APC antibody. **B**) LLC/hEGFR tumors obtained in NSG or B6 mice were evaluated for hEGFR expression.
(TIF)

S4 Fig Glioblastoma cells retain hEGFRt expression in hEGFRt(f/f) mice. hEGFRt expression on GL261-Luc/hEGFRt cells and on tumors obtained after these cells were injected into the CNS of B6 or CAG-hEGFRt(f/f) mice.
(TIF)
